# Kuru and its contribution to medicine

**DOI:** 10.1098/rstb.2008.0070

**Published:** 2008-11-27

**Authors:** D. Carleton Gajdusek

**Affiliations:** Institut Neurologique Alfred Fessard, Centre National de la Recherche ScientifiqueBâtiment 33, Avenue de la Terrasse, 91198 Gif-sur-Yvette, France

**Keywords:** kuru, amyloid, Melanesia

## Abstract

The solution of kuru led us to the solution of Creutzfeldt–Jakob disease and to the elucidation, in humans and other species, of previously unknown mechanisms of infection. These require very close three-dimensional matching, which determines infectious nucleant or prion activity. Evidence for nucleation processes is found widely in the organic and inorganic worlds and in the interactions between them: in the formation of amyloid fibrils; in the biochemistry of silicon; in cave formations deep in the Earth; and in outer space. Kuru in its location in Papua New Guinea has also led to an understanding of the cultural achievements of the Palaeo-Melanesians, with deep roots in human history.

## 1. Kuru

Kuru presented itself to us in 1957 as a challenge to epidemiology and a clearly solvable problem, but it quickly led us to realize that its solution would contribute new vistas to clinical medicine, microbiology, immunology and neurology. It was fast in revealing that it would open new doors to virology, genetics, amyloidology and ageing. Already in 1957, we were calling kuru galloping senescence of the juvenile and soon added to this the key to the ageing brain, amyloidosis, and the genetic control of ageing and the length of human life. It thus behoves me to trace some of the roads down which kuru research has taken us.

Very early we recognized that it presented the problem of an infectious disease with a very long incubation period and its agent appeared to be very small compared with all other viruses, from the high titres it attained in the brain in our initial transmission experiments (Gajdusek 1977). Clinical study of patients revealed quickly that it was non-inflammatory, and laboratory tests and neuropathology confirmed this. Its agent evoked no immune response.

Igor Klatzo initially compared it in 1957 to Creutzfeldt–Jakob disease, which no one had seen in children and was unknown to most British and American physicians; only some 20 cases had been seen in the world. He emphasized that the cerebral amyloid plaques seen in most cases had never before been seen in the brains of children. When J. R. M. Innes and William Hadlow showed us the similarity of the disease to scrapie (Hadlow 1959; Innes & Saunders 1962), we had the lead that led us to repeat our animal inoculations on a much wider basis and proceeded to show that it was transmissible to chimpanzees and to monkeys, rodents and other animals, with long incubation periods.

## 2. Molecular casting: nucleants at nanoscale

We have repeated confirmation of resistance of a portion of infectivity of scrapie to temperature as high as 600°C (Brown *et al.* 2000). The enormous resistance to dry heat of a small fraction (approx. one part in 10^6^) of infectious nucleant activity may represent a molecular cast, or fingerprint, of the nucleant.

Infectious nucleant or prion activity is the result of very close three-dimensional matching. Any particle that can sufficiently mimic the molecule to be nucleated to crystallize, fibrillize or form a two-dimensional molecular sheet can trigger the process. Matching surely must be close enough to evoke van der Waals forces and coulombic forces, even H-bonding.

## 3. Can we preserve biological specificity of antibodies, antigens, receptors, pheromones, transmitters, ion channels or enzymes in inorganic molecular casts or atomic moulds?

The answer is already yes for the first six items and if we allow for synthetic hydrocarbon polymers for molecular casting, it may soon be so for enzymes.

## 4. Dermatoglyphic preserving of biological specificity

One of my adopted New Guinea sons has pointed out to me that a fingerprint using ink from batteries (MnO_2_) is an example of such preservation with no atom of carbon of biological specificity, more individual than the DNA sequence of identical twins.

## 5. Fossils serve for accurate speciation in palaeobotany and palaeozoology

Another of my adopted New Guinea sons has pointed out that fossil footprints serve for accurate taxonomic classifications and yet are not a source of DNA for speciation by polymerase chain reaction. Nucleoli can be counted in inorganic fossils in cells extinct for millions of years.

## 6. Osmium shading in electron microscopy reveals details of molecular structure at nanometric distances

There is no contribution from carbon atoms to the image of an osmium or platinum shade freeze-fracture electron micrograph of individual molecules.

## 7. Mineral nucleants for crystal growth in outer space

Shlichta & MacPherson have sent a series of proteins to outer space to avoid the convection of fluids on the rotating Earth. They nucleate them with a selection of ground minerals. A subset of the minerals usually initiate crystal formation in one or another of the proteins, a differing set for each protein and a differing form of epitactic crystal growth for a given protein with each mineral (McPherson & Shlichta 1988).

## 8. What makes a diamond hold together?

Two polished ancient Chinese copper mirrors applied face to face stick together and require considerable force to separate them.

A stack of newly opened clean microscopic cover-slips slide one upon the other, yet it requires considerable force to separate them.

A diamond is the hardest of minerals, can scratch steel, yet it is still made only of carbon, the same as carbon black, coal or graphite. Sulphur may be pure yet malleable, ductile, fragile or clay like and of many colours depending how the S atoms are packed. Thus, the van der Waals forces are brought into play at atomic distances.

## 9. Twinning of minerals

There are over at least 200 twinning possibilities for quartz (SiO_2_). Most possible forms have been found in the over 30 000 years of search for them. Rare forms are named after the locality where they are first found. When one new twin ‘form’, ‘strain’ or ‘species’ is found, it is usually named after the region where it has been found. Then, it is common to find other examples of this particular strain of twinned quartz in shafts for many hundreds of kilometres around the first finding, but nowhere else on Earth. Much the same is true for gem diamonds, emeralds and rubies and for other examples of twinning in mineralogy.

## 10. Industrial viruses and Ice Nine

‘Ice Nine’ of Kurt Vonnegut Jr in his *Cat's cradle* is a fictional approach to the problem of nucleation (Vonnegut 1963) based on a sound understanding of the phenomenon. His brother, William, was a major meteorologist fully familiar with viruses not containing DNA or RNA and the World War II ethylene diamine tartrate problem of an industrial virus that nucleated the slow appearance of bubbles in large crystals of the compound made for optical purposes. Kurt Vonnegut Jr got the idea correctly.

## 11. Amyloid-enhancing factors are scrapie-like infections/amyloid nucleants

For approximately 35 years, I have been aware of the work of amyloidologists in their attempts to accelerate the appearance of AA amyloid deposits in animals primed with inoculation of AgNO_3_ or heterologous casein. Their discovery of amyloid-enhancing factors (Niewold *et al.* 1987), which were active in high dilutions and difficult to purify, reminded me of our problems with the infectious agents of scrapie or kuru. I suggested that amyloid-enhancing factors were scrapie-like agents (Gajdusek 1988, 1991, 1994*a*,*b*).

## 12. Any β-pleated polymeric assembly as a two-dimensional sheet or as a fibril may act as a heteronucleant for different amyloidogenic proteins

Amyloid deposits in man or animals are always found to be contaminated with other proteins similarly polymerized into fibrils—even co-polymerized. These are all the proteoglycans and glycosaminoglycans as well as plasma P-protein, chymotrypsin, ubiquitin, light chains of gamma globulins and other amyloidogenic proteins.

Tropocollagen is nucleated to fibrillize not only by submicroscopic fibrils of tropocollagen but also by dimers and polymers of glycosaminoglycans (Öbrink 1973) or proteoglycans, and not by heparan, which is a single-bonded dimer.

## 13. Synthesis of prion-like infectious nucleants

Katarzyna Lundmark and Per Westermark have succeeded in getting *in vivo* heterologous nucleation of beta-fibrillary protein polymerization into amyloid fibrils with synthetic amyloid-enhancing factors (Johan *et al.* 1998). Such heterologous nucleants have been synthetic peptides from the highly fibrillogenic section of the amyloid precursor protein of both transthyretin- and insulin-associated amyloid. They serve to nucleate the fibrillization of AA amyloid precursor protein when polymerized into small fibrils, but not as unpolymerized peptides. Labelling with I^131^ has served to locate AA amyloid fibrils that have been nucleated by these heterologous amyloid-enhancing factors. Thus, if these replicating systems are thought to be alive, they have already synthesized ‘life’ and published it.

Per Westermark and his colleagues have demonstrated the induction of AA amyloidogenesis by nucleation with heteronucleants such as silk (Lundmark *et al.* 2005) and spider webs and oral paté de foie gras in transgenic mice (Solomon *et al.* 2007).

## 14. Nucleation in speleology

In exploring caves, speleologists are familiar with nearly identical formations surrounding a fallen and shattered stalagmite or stalactite, very much like the ring of small mushrooms around an old large mushroom on the forest floor or the ring of young sequoia saplings around a dead trunk. They often resemble even the odd idiosyncrasies of the ‘parent’ formation.

At times a cavern of brown ‘toadstools’ or one of pink ‘phallic’ organ-pipe cactuses is filled with dozens or hundreds of uncannily similar replicas. Then several galleries below the dark brown ‘toadstools’, in a gallery of the pink ‘phalluses’, stands a dark brown toadstool from a nucleant that has tumbled down millions of years ago to the lower gallery from the ‘toadstool’ room above.

## 15. Biological macromolecules all interact strongly with SiO_2_ containing the most common elements on the surface of the Earth

Iler (1977) and Weiss (1981) have shown how silicon and oxygen in the form of SiO_2_ can interact with and bond to biological macromolecules or polymers in long series of strong attractions, whether they are carbohydrates, proteins, nucleic acids or fats. These are the two most common elements on the surface of the Earth. The role of silicon in this is fully discussed in the Nobel Foundation's *The biochemistry of silicon* *and related problems*, in which Iler's article appeared. Thus, binding to solid is the most likely origin of life, not primordial oceanic liquid.

## 16. Montmorillonite clay deposits lead to delayed neurodegenerative diseases

The high-incidence foci of two very different diseases, Guamanian amyotrophic lateral sclerosis (*lytico*) and parkinsonism–dementia (*bodig*), occurred also in a few remote inland villages on Honshu Island in Japan and among the Auyu and Jakai people around Bade and Kepi in southern West New Guinea (Gajdusek & Salazar 1982). It has virtually disappeared from all of these places with the introduction of civilization. These three foci were restricted to remote communities in which there was such a depletion of environmental calcium as to produce a chronic severe deficiency of calcium in the diet, with the result that calcium sparing led to soft tissue deposition of calcium aluminium silicate or montmorillonite clay deposits within brain cells, along with other heavy elements as the diet provided. These lay dormant for decades until triggered later in life to cause specific neuronal damage leading to *lytico* or *bodig*. Civilization, with all its ills, has caused these two diseases in all the three locations to disappear.

## 17. Nucleation in extragalactic space

The odd patterns of distant galaxies of billions of stars is well known to all amateur astronomers who have viewed in awe the thousands of photographs we now have of them. They are by no means random patterns of stars, but lend rather easily to classification by similar appearance, as do most patterns in geophysics. These similar patterns appear in groups and in a very small fraction of the 2*π* steradians of space around us. This is cause for wonder about nucleation of such patterns across distances of huge numbers of light years.

## 18. Conclusion

In pursuing the establishment of the boundary of kuru through many different cultures and linguistic groups, we discovered that kuru had no genetic boundaries. We also discovered many languages of the Kukukuku people who formed its southeastern boundary. With them quickly revealing to us extreme differences in their pattern of psychosexual development and initiations by age grade, I was led by this to the concept of Palaeo-Melanesian (Papuan) cultures in which I found traits of mime, myth, music and metaphor characterizing human culture as apart from all other animals. These were all speakers of Papuan (non-Austronesian) languages. Archaeological and genetic findings have confirmed that there is a depth of culture going back at least 30 000 years in Melanesia; it is not part of the much later Polynesian explosion into the Pacific, which dates back only 3500 years. The discovery that Palaeo-Melanesian cultures had no chieftainship, no class and caste, no peasantry or markets, no slavery, torture or prisons and no priests or shamans has revolutionized sociological thinking and left us in awe of the Palaeo-Melanesians.
